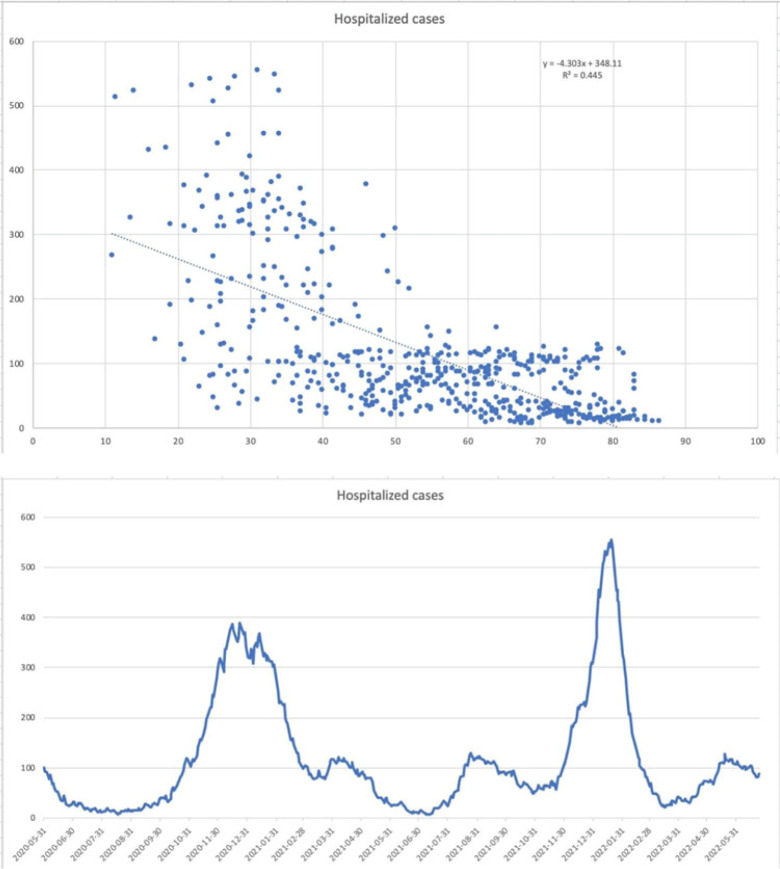# Outcomes of patients hospitalized for COVID-19, secondary infections, antimicrobial use during SARS-CoV-2 delta and omicron variants

**DOI:** 10.1017/ash.2023.299

**Published:** 2023-09-29

**Authors:** Swetha Srialluri, Curtis Collins, Holly Murphy

## Abstract

**Background:** The SARS-CoV-2 omicron variant has been associated with increased transmissibility and less severe disease than the SARS-CoV-2 delta variant. Low rates of secondary infections and excess empiric antimicrobial use were reported early in the pandemic. Comparisons between later variants are not as well documented. We evaluated outcomes for SARS-CoV-2 delta- and omicron-variant surges with emphases on COVID-19–related treatment, secondary infections, and antimicrobial utilization. **Methods:** A single-center, observational, retrospective study was conducted for SARS-CoV-2–positive patients admitted to our 548-bed community teaching hospital between November and December 2021 (SARS-CoV-2 delta-variant–predominant phase) and January–February 2022 (SARS-CoV-2 omicron-variant–predominant phase). Demographic and outcome data were obtained from the institutional data warehouse and were compared between groups. Secondary infections were defined as positive blood and respiratory culture results during admission, with likely contaminants excluded. Mann-Whitney *U* tests were used to evaluate continuous variables, and *t* tests were used to analyze categorical variables. *P* ≤ .05 was considered statistically significant. **Results:** In total, 1,297 patients were included: 787 (60.7%) in SARS-CoV-2 delta-variant–predominant phase and 510 (39.3%) in SARS-CoV-2 omicron-variant–predominant phase. Patients in SARS-CoV-2 omicron-variant–predominant phase were more often vaccinated (37.7% vs 55%; *P* < .001), required lower rates of ICU care (16.0% vs 11.6%; *P* = .025), and required less intubation (13% vs 6.3%; *P* < .001). Utilization of remdesivir (51.0% vs 32.2%; *P* < .001), dexamethasone (70.8% vs 43.3%; *P* < .001), and tocilizumab or baricitinib (14.5% vs 5.3%; *P* < .001) decreased during the SARS-CoV-2 omicron-variant–predominant phase. Length of stay (5 days vs 4 days; *P* < .001) and 30-day mortality also decreased during this period (16.40% vs 9.8%; *P* = .001). Infectious diseases consultation increased during the SARS-CoV-2 omicron-variant–predominant phase (39.8% vs 45.5%; *P* = .042). There was no significant difference in patients with positive blood cultures (3.4% vs 1.8%; *P* = .074), but there was a significant decrease in positive respiratory cultures (5.8% vs 2.7%; *P* = .009), combining for an overall reduction (8.4% vs 4.1%; *P* = .003). The incidence of overall antimicrobial use increased during the omicron-predominant phase (36.1% vs 41.8%; *P* = .04), and duration was lower (5 days vs 4 days; *P* < .001). Antimicrobial class-specific duration was unchanged, with the exception of decreased gram-positive agents (3 days vs 2 days; *P* = .012). **Conclusions:** Our results confirm previous reports of reduced disease severity during the SARS-CoV-2 omicron-variant–predominant period. The incidence of secondary infections decreased, driven by a reduction in respiratory infections. Antimicrobials were used at increased rates and for shorter durations during the SARS-CoV-2 omicron-variant–predominant period.

**Disclosures:** None

**Figure:**